# Impact of endometrial preparation on early pregnancy loss and live birth rate after frozen embryo transfer: a large multicenter cohort study (14 421 frozen cycles)

**DOI:** 10.1093/hropen/hoac007

**Published:** 2022-02-15

**Authors:** L Vinsonneau, J Labrosse, G Porcu-Buisson, N Chevalier, J Galey, N Ahdad, J P Ayel, C Rongières, P E Bouet, E Mathieu d’Argent, I Cédrin-Durnerin, F Pessione, N Massin

**Affiliations:** Department of Reproductive Medicine, Hopital Tenon, Paris, France; Department of Reproductive Medicine and Fertility Preservation, CHU Jean-Verdier, Bondy, France; Department of Reproductive Medicine, Institut de Médecine de la Reproduction, Marseille, France; Department of Reproductive Medicine, Polyclinique Saint-Roch, Montpellier, France; Department of Reproductive Medicine, Institut Montsouris, Paris, France; Department of Reproductive Medicine, Hopital Tenon, Paris, France; Department of Reproductive Medicine, Grand Hôpital de l'Est Francilien, Meaux, France; Department of Reproductive Medicine, Groupe Hospitalier Diaconesses Croix Saint-Simon, Paris, France; Department of Reproductive Medicine, Centre Médico-Chirurgical Obstétrique, Strasbourg, France; Department of Reproductive Medicine, CHU Angers, Angers, France; Department of Reproductive Medicine, Hopital Tenon, Paris, France; Department of Reproductive Medicine and Fertility Preservation, CHU Jean-Verdier, Bondy, France; Department of Procreation, Embryology and Human Genetics, Agence de la Biomédecine, Paris, France; Department of Reproductive Medicine, Intercommunal Hospital—University Paris Est, Créteil, France

**Keywords:** miscarriage, HRT, embryo transfer, natural cycle, IVF/ICSI outcome

## Abstract

**STUDY QUESTION:**

Does the endometrial preparation protocol (artificial cycle (AC) vs natural cycle (NC) vs stimulated cycle (SC)) impact the risk of early pregnancy loss and live birth rate after frozen/thawed embryo transfer (FET)?

**SUMMARY ANSWER:**

In FET, ACs were significantly associated with a higher pregnancy loss rate and a lower live birth rate compared with SC or NC.

**WHAT IS KNOWN ALREADY:**

To date, there is no consensus on the optimal endometrial preparation in terms of outcomes. Although some studies have reported a higher pregnancy loss rate using AC compared with NC or SC, no significant difference was found concerning the pregnancy rate or live birth rate. Furthermore, no study has compared the three protocols in a large population.

**STUDY DESIGN, SIZE, DURATION:**

A multicenter retrospective cohort study was conducted in nine reproductive health units in France using the same software to record medical files between 1 January 2012 and 31 December 2016. FET using endometrial preparation by AC, modified NC or SC were included. The primary outcome was the pregnancy loss rate at 10 weeks of gestation. The sample size required was calculated to detect an increase of 5% in the pregnancy loss rate (21–26%), with an alpha risk of 0.5 and a power of 0.8. We calculated that 1126 pregnancies were needed in each group, i.e. 3378 in total.

**PARTICIPANTS/MATERIALS, SETTING, METHODS:**

Data were collected by automatic extraction using the same protocol. All consecutive autologous FET cycles were included: 14 421 cycles (AC: n = 8139; NC: n = 3126; SC: n = 3156) corresponding to 3844 pregnancies (hCG > 100 IU/l) (AC: n = 2214; NC: n = 812; SC: n = 818). Each center completed an online questionnaire describing its routine practice for FET, particularly the reason for choosing one protocol over another.

**MAIN RESULTS AND THE ROLE OF CHANCE:**

AC represented 56.5% of FET cycles. Mean age of women was 33.5 (SD ± 4.3) years. The mean number of embryos transferred was 1.5 (±0.5). Groups were comparable, except for history of ovulation disorders (*P* = 0.01) and prior delivery (*P* = 0.03), which were significantly higher with AC. Overall, the early pregnancy loss rate was 31.5% (AC: 36.5%; NC: 25.6%; SC: 23.6%). Univariable analysis showed a significant association between early pregnancy loss rate and age >38 years, history of early pregnancy loss, ovulation disorders and duration of cryopreservation >6 months. After adjustment (multivariable regression), the early pregnancy loss rate remained significantly higher in AC vs NC (odds ratio (OR) 1.63 (95% CI) [1.35–1.97]; *P* < 0.0001) and in AC vs SC (OR 1.87 [1.55–2.26]; *P* < 0.0001). The biochemical pregnancy rate (hCG > 10 and lower than 100 IU/l) was comparable between the three protocols: 10.7% per transfer.

**LIMITATIONS, REASONS FOR CAUTION:**

This study is limited by its retrospective design that generates missing data. Routine practice within centers was heterogeneous. However, luteal phase support and timing of embryo transfer were similar in AC. Univariable analysis showed no difference between centers. Moreover, a large number of parameters were included in the analysis.

**WIDER IMPLICATIONS OF THE FINDINGS:**

Our study shows a significant increase in early pregnancy loss when using AC for endometrial preparation before FET. These results suggest either a larger use of NC or SC, or an improvement of AC by individualizing hormone replacement therapy for patients in order to avoid an excess of pregnancy losses.

**STUDY FUNDING/COMPETING INTEREST(S):**

The authors declare no conflicts of interest in relation to this work. G.P.-B. declares consulting fees from Ferring, Gedeon-Richter, Merck KGaA, Theramex, Teva; Speaker’s fees or equivalent from Merck KGaA, Ferring, Gedeon-Richter, Theramex, Teva. N.C. declares consulting fees from Ferring, Merck KGaA, Theramex, Teva; Speaker’s fees or equivalent from Merck KGaA, Ferring. C.R. declares a research grant from Ferring, Gedeon-Richter; consulting fees from Gedeon-Richter, Merck KGaA; Speaker’s fees or equivalent from Merck KGaA, Ferring, Gedeon-Richter; E.M.d’A. declares Speaker’s fees or equivalent from Merck KGaA, MSD, Ferring, Gedeon-Richter, Theramex, Teva. I.C-D. declares Speaker’s fees or equivalent from Merck KGaA, MSD, Ferring, Gedeon-Richter, IBSA. N.M. declares a research grant from Merck KGaA, MSD, IBSA; consulting fees from MSD, Ferring, Gedeon-Richter, Merck KGaA; Speaker’s fees or equivalent from Merck KGaA, MSD, Ferring, Gedeon-Richter, Teva, Goodlife, General Electrics.

**TRIAL REGISTRATION NUMBER:**

N/A.

WHAT DOES THIS MEAN FOR PATIENTS?

There are three different approaches that can be used to prepare the uterus before frozen embryo transfer. These are known as the artificial cycle, the natural cycle and the stimulated cycle. To date, there is no consensus on which approach should be used. Some studies have reported that artificial cycle led to higher pregnancy loss rates, but no clear difference in pregnancy rate or live birth rate has been established. Furthermore, no study has compared the three approaches in a large population.

This study analyzed 14 421 cycles of frozen embryo transfer, corresponding to 3844 pregnancies. It shows that the artificial cycle is associated to significantly higher early pregnancy loss rates and significantly lower live birth rates compared to the other approaches. These results suggest either a greater use of the other approaches (natural cycle or stimulated cycle), or that the artificial cycle protocol should be improved.

## Introduction

Since the first birth resulting from frozen embryo transfer (FET) in 1984 in Australia, the number of FET cycles has progressively increased. The practice of FET has been enhanced by significant improvements in the field of cryopreservation (vitrification) and by the development of elective single embryo transfers to limit multiple pregnancies (de Mouzon *et al.*, 2020). In Europe in 2016, FET accounted for 44.1% of all transfers (Wyns *et al.*, 2020). Obstetrical and perinatal outcomes of pregnancies after FET are reassuring (Wong *et al.*, 2017). The proportion of children born after FET is increasing. In France in 2017, FET represented 29.2% of live births obtained with ART and 26.3% in Europe in 2014 (‘Agence de la Biomédecine,’ n.d.).

The success of FET relies on embryo quality, uterine receptivity and synchronization between the endometrium and the embryo. FET should be performed at a time when the endometrium is the most receptive, known as the ‘implantation window’. Three types of endometrial preparation protocols are used in current practice. The artificial cycle (AC) consists in exogenous supplementation by estradiol and progesterone. It is the most commonly used protocol worldwide as it enables flexibility and regulation of embryo transfers for IVF centers. Stimulated cycle (SC) consists in ovarian stimulation by exogenous treatments (gonadotropins, letrozole or clomifene citrate for example) followed by ovulation triggering by hCG. The natural cycle (NC), which is increasingly used, consists in the monitoring of a physiological cycle. It can be ‘modified’ by using hCG to trigger ovulation and/or associated to a luteal phase support by progesterone.

Previous studies have reported that the early pregnancy loss rate in FET was higher when using the AC protocol compared to NC or SC (Veleva *et al.*, 2008; Tomás *et al.*, 2012; Cerrillo *et al.*, 2017; Peigné *et al.*, 2019; Liu *et al.*, 2020; Levi Setti *et al.*, 2020). A 7-year study of vitrified blastocyst transfers found that the risk of early pregnancy loss was higher using AC compared to NC or modified NC with ovulation triggering (Levi Setti *et al.*, 2020). Similarly, a retrospective study of 1846 normally ovulating patients also reported that the early pregnancy loss rate was significantly higher with AC than with NC (Liu *et al.*, 2020). However, so far, no consensus exists concerning the use of one protocol over another. Furthermore, none of these protocols has shown its superiority in terms of live birth rate (Groenewoud *et al.*, 2013; Ghobara *et al.*, 2017).

The aim of our study was to determine in a large cohort whether the type of protocol for endometrial preparation has an impact on the risk of early pregnancy loss and live birth rate after FET, as well as the risk factors associated to early pregnancy loss after FET.

## Materials and methods

### Design

Our study is a multicenter retrospective study using data collected prospectively from January 2012 to December 2016. Participating centers were selected if they used the same electronic health record software (MediFirst-AMP^®^) and if the three types of endometrial preparation protocols were performed.

### Data collection and ethics

Data were obtained from registries of nine French IVF centers: Angers, Bondy, Creteil, Marseille, Montpellier, Strasbourg and three in Paris (Diaconesses, Institut Mutualiste Montsouris, Tenon). In each center, the registry was continuously updated using MediFirst-AMP^®^ (www.medifirst.net), with a format that meets requirements of the French Biomedicine Agency (Agence de la Biomédecine). The use of data from these registries for purposes of observational studies complies with national regulations (French Data Protection Authority; Commission Nationale de l’Informatique et des Libertés or CNIL). According to French law (2012-300), patients are aware that their data can be used for anonymous clinical studies unless they specifically object. Individual registries were integrated into a global database and processed in full conformity with the reference methodology (MR-003) of the French Data Protection Authority (CNIL 2016). Data on each frozen cycle of each center were merged in a final database. For each cycle, baseline characteristics, treatment-related data and reproductive outcomes were reported up to live birth. Data were included for all cycles performed between 2012 and 2016. According to French law, approval of an ethical committee is not required for research on previously collected data.

An online questionnaire was also sent to each participating center to collect information on routine practice regarding endometrial preparation before FET (choice of preparation, patient profile, type of drugs used, etc.).

### Endometrial preparation protocols

In AC protocols, estrogen supplementation was performed by using Provames^®^ (between 4 mg and 6 mg by oral route or vaginal route) and Vivelledot^®^ (skin patch 100 μg ± 50 μg every 2–4 days). Three centers performed prior hypophyseal block by injection of a GnRH agonist (Decapeptyl^®^ 3.75 mg) after checking endometrial thickness by ultrasound. Natural micronized progesterone (Utrogestan^®^ or Progestan^®^) was introduced vaginally, between 400 mg and 800 mg, depending on the center. FET was performed after 2 or 3 days of progesterone for Day 2 or Day 3 embryos and after 5 days of progesterone for blastocysts. Administration of progesterone was continued until at least 9 weeks of gestation in all centers.

All NC were modified NC protocols. Modified NC protocols had luteal phase support by progesterone. According to centers, ovulation was triggered by recombinant hCG. In modified NC, monitoring by ultrasound and hormone assay (estrogen, LH and progesterone serum levels) was performed between Day 9 and Day 12.

In SC, recombinant or urinary gonadotropin (50–75 IU) was used and ovulation was triggered when a dominant follicle (>16–17 mm) and an adequate endometrial thickness were present on ultrasound. Luteal phase support by progesterone was added.

### Endpoints

The main endpoint was the early pregnancy loss rate before 10 weeks of gestation among the total number of pregnancies (defined by hCG > 100 IU/ml). Secondary endpoints were the pregnancy rate per FET (number of pregnancies with hCG > 100 IU/ml/number of FET), the biochemical pregnancy rate per pregnancy (number of pregnancies with hCG > 10 and lower than 100 IU/l/number of pregnancies) and the live birth rate per FET (number of liveborn infants after 22 weeks of gestation/number of FET).

### Inclusion/exclusion criteria

Criteria for inclusion of patients were age between 18 and 43 years and at least one frozen/thawed embryo transfer performed, whatever the embryo stage and technique used (IVF or ICSI). Exclusion criteria were gamete donation, the use of sperm obtained by surgical extraction and preimplantation genetic testing.

### Calculation of the number of subjects needed

The average rate of early pregnancy loss in FET is 25% (‘Agence de la Biomédecine,’ n.d.). To show a significant increase of 5% with AC compared with NC or SC (21% vs 26%) with an alpha risk of 5% and a power of 80%, it was necessary to analyze 1126 pregnancies per group, i.e. a total of 3378 pregnancies obtained after FET. Given the pregnancy rate after FET (26%), it was necessary to include a total of ∼ 13 000 FET cycles.

### Statistical analyses

Qualitative variables are described in numbers and percentages. Proportions were compared using Chi-squared tests. Continuous variables were compared by ANOVA. Continuous variables were included in univariable or multivariable analysis in the form of continuous variables or clinically relevant classes.

Logistic regression analysis was used to identify factors associated with the risk of early pregnancy loss. Variables were analyzed according to their relevance or to the degree of significance in the different comparative analyses performed. Variables associated with the risk of early pregnancy loss in univariable analysis with a *P* ≤ 0.20 were selected for inclusion in the multivariable model. The threshold of significance was defined by a *P-*value ≤ 0.05. Statistical analyses were performed using SAS software version 9.4.

## Results

**Figure 1.**
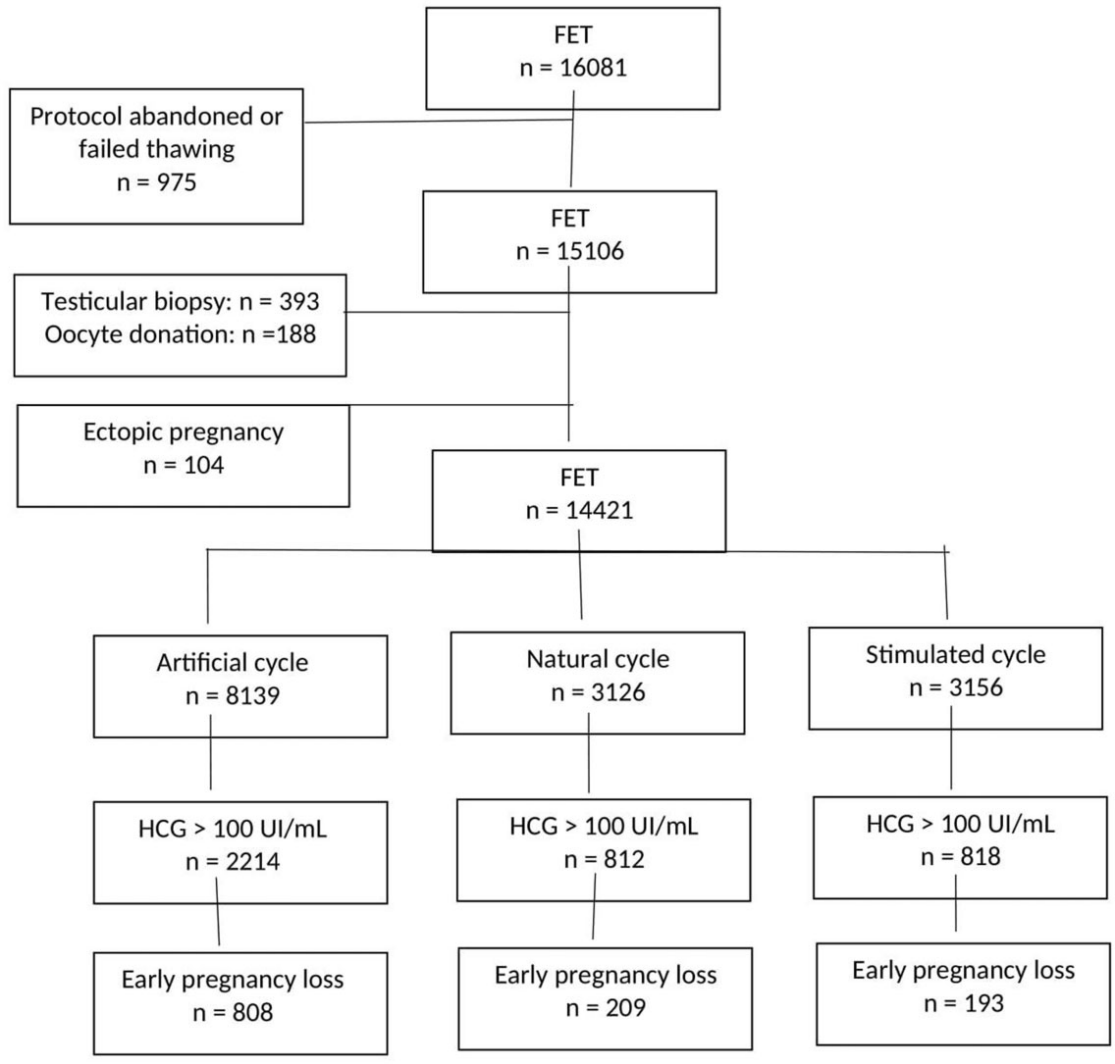
Flow chart of frozen embryo transfer (FET), endometrial preparation protocols and early pregnancy loss.

We collected data from 16 081 FET cycles. In total, 14 421 cycles comprising 3844 pregnancies (hCG > 100 IU/ml) were included in the study. No patient underwent more than one cycle. Ectopic pregnancies (n = 104, 2.6%) were excluded (1.4% in AC, 0.6% in NC and 0.4% in SC) (Fig. 1). Patient characteristics and number of frozen–thawed embryo transfers according to endometrial preparation protocol are shown in Supplementary Table SI.

In the whole study population, the live birth rate after FET was 17.8% and the pregnancy rate after FET was 26.6%. The early pregnancy loss rate was 31.5% (range: 27.3–35.1%) and did not statistically differ between centers (*P* = 0.1). No significant difference was found between the years during which FET were performed (2012, 2013, 2014, 2015 and 2016) (*P* = 0.63). Distribution of cycles, pregnancies and early pregnancy loss according to the three protocols is shown in Fig. 1.

### Use of endometrial preparation protocols according to the study center

Overall, AC was the most common protocol (56.5% of cycles) and was used first line in five of the nine study centers. SC and NC were used to a similar degree (21.8% vs 21.7%, respectively).

NC was used first line by three centers. All centers used modified NC. SC was used first line in two centers. In NC and SC, FET was performed according to hormonal monitoring and embryo stage (Day 2, Day 3 or blastocysts) (Labrosse *et al.*, 2020).

For all FET, blood samples to measure serum hCG levels were performed at Days 12, 14 and 16 after embryo transfer. Vitrification was the method of freezing used for all embryos.

### Outcomes of FET cycles

Results are reported in Table I. The early pregnancy loss rate was significantly higher for AC in comparison with NC and SC (36.5% vs 25.7% vs 23.6%, respectively, *P* < 0.005). The early pregnancy loss rate was also significantly higher for NC compared with SC, but the threshold of significance was lower (*P* = 0.03). The live birth rate was significantly lower for AC compared with NC [odds ratio (OR): 0.88 [0.79–0.98] (95% confidence interval)] and SC [OR: 0.85 [0.76–0.94] (95% confidence interval)] (16.9% vs 18.8% vs 19.3%, respectively, *P* < 0.003). No difference between the different types of protocol was found in terms of biochemical pregnancies (hCG > 10 and lower than 100 IU/l) and rates of pregnancies with hCG > 100 IU/ml.

**Table I:** Patient characteristics and outcomes of frozen–thawed embryo transfer according to endometrial preparation protocol.

	Total population	Artificial cycle (AC)	Natural cycle (NC)	Stimulated cycle (SC)	*P*
Number of FET	14 421	8139	3126	3156	
Age (years), mean ± SD	34.2	34.1 ± 4.7	34.3 ± 4.5	34.2 ± 4.4	0.70
Primary infertility, n (%)	7326 (50.8)	4182 (51.4)	1513 (48.4)	1541 (48.8)	0.004
Length of infertility (days), mean ± SD	2042 ± 1054	2054 ± 1,059	2015 ± 1004	2035 ± 1088	0.92
History of early pregnancy loss, n (%)	2064 (14.3)	1134 (13.9)	476 (15.2)	454 (14.4)	0.21
BMI (kg/m^2^), mean ± SD	23.4 ± 5.4	23.8 ± 5.2	22.7 ± 5.8	23.6 ± 5.2	0.07
Smoking, n (%)	3390 (23.5)	1923 (23.6)	736 (23.5)	731 (23.2)	0.87
ICSI, n (%)	8321 (57.7)	4638 (57.0)	2036 (65.1)	1647 (52.2)	<0.0001
Endometrial thickness (mm), mean ± SD	9.3 ± 2.5	9.1 ± 2.6	9.8 ± 2.3	9.1 ± 2.2	0.02
Number of embryos transferred, mean ± SD	1.4 ± 0.5	1.4 ± 0.5	1.3 ± 0.5	1.5 ± 0.5	0.09
Biochemical pregnancy (hCG 10–100 IU/ml) per pregnancy, n (%)	461 (10.7)	251 (10.2)	95 (10.5)	115 (12.2)	0.20
Pregnancy with HCG >100 IU/ml per FET, n (%)	3844 (26.7)	2214 (27.2)	812 (26.0)	818 (25.9)	0.20
Early pregnancy loss per pregnancy with HCG >100 IU/ml, n (%)	1210 (31.5)	808 (36.5)	209 (25.7)	193 (23.6)	<0.005
Live birth per FET, n (%)	2571 (17.8)	1375 (16.9)	588 (18.8)	608 (19.3)	<0.003

FET, frozen embryo transfer.

### Risk factors for early pregnancy loss: logistic regression

Characteristics of patients having obtained a pregnancy with hCG >100 IU/ml are shown in Table II. Patients were comparable between the three groups, except for higher BMI and more patients with ovulation disorder in AC and SC. These differences are related to indications reported by the centers (NC was not used for patients with polycystic ovary syndrome). A history of childbirth was significantly more frequent in the AC group and endometrial thickness was significantly greater in the NC group.

**Table II:** Characteristics of patients with pregnancy with HCG >100 IU/ml after FET according to endometrial preparation protocol.

	Total cycles	Artificial cycle (AC)	Natural cycle (NC)	Stimulated cycle (SC)	*P*
Number of pregnancies with HCG >100 IU/ml	3844	2214	812	818	
Age (years), mean ± SD	33.5 ± 4.3	33.3 ± 4.4	33.7 ± 4.3	33.8 ± 4.2	0.40
BMI (kg/m^2^), mean ± SD	21.9 ± 4.8	22.4 ± 4.5	21.4 ± 5.1	21.1 ± 5.6	0.01
Smoking (%)	879 (22.9)	517 (23.4)	188 (23.2)	174 (21.3)	0.50
Ovulation disorder* (%)	937 (24.4)	682 (30.8)	89 (11)	166 (20.3)	0.01
History of early pregnancy loss (%)	591 (13.4)	335 (15.1)	131 (16.1)	125 (15.3)	0.80
History of childbirth (%)	1919 (49.9)	1137 (51.5)	373 (48.4)	409 (48.8)	0.03
Uterine disease (%)	261 (6.8)	152 (6.9)	65 (8)	44 (5.4)	0.10
Endometriosis (%)	383 (10)	228 (10.3)	69 (8.5)	86 (10.5)	0.30
Endometrial thickness (mm), mean ± SD	9.3 ± 2.6	9 ± 2.8	9.9 ± 2.4	9.2 ± 2.2	<0.01
Number of embryos transferred, mean ± SD	1.4 ± 0.5	1.4 ± 0.5	1.3 ± 0.5	1.5 ± 0.5	0.09
Duration of freezing (days), mean ± SD	363 ± 470	354 ± 467	382 ± 465	371 ± 484	0.60

* Ovulation disorders include central anovulation, polycystic ovary syndrome and primary ovarian insufficiency.

FET, frozen embryo transfer.

A logistic regression model with adjustment was used to identify factors significantly correlated with the risk of early pregnancy loss. Results of the univariable and multivariable analyses are shown in Table III. Smoking and BMI were included in the multivariable analysis despite the lack of significance in univariable analysis, as previous studies have shown these variables to be associated with the risk of early pregnancy loss (Boots and Stephenson, 2011; Pineles *et al.*, 2014; Cavalcante *et al.*, 2019; Ghimire *et al.*, 2020). After adjustment for confounding variables (age, BMI, smoking, history of childbirth or of early pregnancy loss, ovulation disorder, endometriosis, uterine disease and date of embryo freezing), the multivariable analysis showed a greater risk of early pregnancy loss with AC compared with NC, with an OR of 1.63 (1.35–1.97; *P* < 0.0001). There was no significant difference between NC and SC. Other factors significantly associated to the risk of early pregnancy loss after FET were maternal age over 38 years at the time of embryo freezing, history of early pregnancy loss, ovulation disorder and length of embryo storage superior to 6 months. Early pregnancy loss per pregnancy with hCG >100 IU/ml according to centers is shown in Supplementary Table SII.

**Table III:** Risk of early pregnancy loss per pregnancy with HCG >100 IU/ml according to endometrial preparation protocol in univariable and multivariable analysis.

	Early pregnancy loss		Univariable analysis	Multivariable analysis	
	N	%	Odds ratio (CI 95%)	Odds ratio (CI 95%)	*P*
**Endometrial preparation protocol**					
Natural cycle	209	25.7	1	1	–
Stimulated cycle	193	23.6	0.89 (0.71–1.12)	0.88 (0.69–1.10)	0.26
Artificial cycle	808	36.5	1.66 (1.38–1.78)	1.63 (1.35–1.97)	<0.0001
**Age** **(years)**					
25–34	626	28.9	1	1	–
<25	27	37.5	1.48 (0.91–2.41)	1.44 (0.88–2.37)	0.15
35–37	264	30.3	1.07 (0.90–1.27)	1.05 (0.88–1.25)	0.62
38–40	204	38	1.51 (1.24–1.84)	1.50 (1.22–1.84)	0.0001
>40	89	45.9	2.09 (1.55–2.81)	2.01 (1.48–2.74)	<0.0001
**BMI (kg/m^2^)**					
18–24	687	31.2	1	1	–
<18	45	37.2	1.30 (0.89–1.91)	1.34 (0.91–1.98)	0.14
25–29	210	31.4	1.01 (0.84–1.22)	1.04 (0.85–1.26)	0.72
≥30	125	33.7	1.12 (0.89–1.41)	1.09 (0.85–1.39)	0.49
Missing data	143	29.5	0.92 (0.74–1.14)	1.32 (0.97–1.79)	0.08
**Smoking**					
No	779	31.6	1	1	–
Yes	295	33.6	1.09 (0.93–1.29)	1.13 (0.95–1.34)	0.16
Missing data	136	27.4	0.82 (0.66–1.01)	0.88 (0.63–1.19)	0.37
**History of early pregnancy loss**					
No			1	1	
Yes	277	22.9	2.19 (1.83–2.62)	2.13 (1.72–2.62)	<0.0001
Ovulation disorder*					
No	738	30.1	1	1	–
Yes	342	36.5	1.33 (1.14–1.56)	1.23 (1.04–1.45)	0.01
Missing data	130	28.4	0.92 (0.74–1.14)	1.10 (0.82–1.48)	0.52
**Uterine disease**					
No	1108	30.9	1	1	
Yes	102	39.1	1.43 (1.11–1.86)	1.20 (0.91–1.57)	0.19
**Endometriosis**					
No	1069	30.9	1		
Yes	141	36.8	1.30 (1.05–1.62)	1.24 (0.99–1.56)	0.07
**Length of embryo storage**					
<3 months	272	27.4	1	1	–
3–6 months	368	32.5	1.27 (1.05–1.53)	1.20 (0.99–1.45)	0.06
6–12 months	240	34.2	1.37 (1.14–1.69)	1.29 (1.04–1.60)	0.02
>12 months	326	32.4	1.26 (1.04–1.53)	1.24 (1.01–1.51)	0.04
Missing data	4	36.4	1.51 (0.44–5.20)	1.22 (0.34–4.32)	0.76
**Embryo stage**					
Day 2	164	35.3	1	1	–
Day 3	499	30.4	0.80 (0.64–0.99)	0.83 (0.66–1.05)	0.11
Blastocyst	543	31.5	1.00 (0.87–1.15)	0.88 (0.70–1.11)	0.28
Zygote	4	28.6	0.73 (0.23–2.37)	0.71 (0.21–2.37)	0.57

* Ovulation disorders include central anovulation, polycystic ovary syndrome, and primary ovarian insufficiency.

## Discussion

This multicenter cohort study of 14 421 FET cycles and 3844 pregnancies shows that endometrial preparation by AC is associated to a significantly higher risk of early pregnancy loss and significantly lower live birth rate compared with NC and SC. The rate of pregnancies with hCG >100 IU/ml was comparable between the three types of protocols.

To our knowledge, our study is the first to compare the early pregnancy loss rate between the three types of endometrial preparation protocols for FET. So far, no consensus exists on which protocol leads to the best pregnancy rates and clinical outcomes (Groenewoud *et al.*, 2013; Peeraer *et al.*, 2015; Yu *et al.*, 2015; Cerrillo *et al.*, 2017; Ghobara *et al.*, 2017). This lack of significant difference between protocols may relate to insufficient sample sizes and an overall low level of proof. The power of our study was high, as it included a large number of FET cycles and considered various parameters affecting the likelihood of childbirth. However, the fact that the AC group comprised more patients than the NC and SC group (n = 2214 vs 812 vs 818, respectively) might limit the validity of our findings. Furthermore, the AC group comprised a significantly higher proportion of patients with ovulatory disorders (31% for AC vs 11% for NC vs 20% for SC, respectively, *P* < 0.01), which is explained by the fact that endometrial preparation by AC was more prescribed to patients with ovulatory disorders such PCOS, central anovulation and primary ovarian insufficiency compared to NC and SC protocols. Since PCOS patients are associated to higher risks of early pregnancy loss, this fact may also increase the proportion of pregnancy losses in the AC group compared to the other groups. Moreover, the risk of early pregnancy loss considering clinical pregnancies has not been evaluated, as cut-offs and definitions used in our study were determined in accordance with recommendations of the French Biomedicine Agency (Agence de la Biomédecine). Further studies using more standardized definitions are warranted (Kolte *et al.*, 2015). After adjustment on potential risk factors, we found that the different types of protocol were similar in terms of biochemical pregnancies (hCG > 10 and lower than 100 IU/l) and rates of pregnancies with hCG >100 IU/ml. Hence, defective implantation might not be the only explanation of the increased risk of early pregnancy loss with AC, which has a significant impact on the likelihood of childbirth. Altogether, the exact mechanisms underlying the enhanced rates of early pregnancy losses reported in our study remain to be established. Although AC was significantly associated to lower rates of live birth compared to NC and SC (16.9% vs 18.8% vs 19.3%, respectively, *P* < 0.05), the variation among the three groups was not as distinct as the variation observed for early pregnancy loss.

These results are in line with findings of several earlier and smaller studies. The randomized prospective study of Cerrillo *et al.* led in 2011–2012 on 570 FET cycles found a first-trimester early pregnancy loss rate of 21.2% with AC, 12.9% with NC and 11.1% with modified NC (*P* = 0.01) (Cerrillo *et al.*, 2017). These rates are lower than those reported in our study but exclusion criteria differed: patients were under 39 years of age and had regular cycles, and patients with stage III/IV endometriosis and polycystic ovary syndrome were excluded. In their retrospective study of 4470 FET, Tomás *et al.* (2012) also reported a higher rate of early pregnancy loss with AC compared with modified NC and NC with luteal phase support by progesterone (41.5% vs 33.6% vs 22.4%, respectively, *P* < 0.001). In a retrospective analysis of 1132 FET, Veleva *et al.* (2008) showed that the early pregnancy loss rate was 1.7 times higher in AC than in the NC and fresh embryo transfer cycles. A more recent French retrospective study analyzing 1926 FET also found an increase of over 20% of early pregnancy loss with AC compared with SC (Hatoum *et al.*, 2018). Live birth rate was also significantly lower with AC (29.6%) than SC (29.6% vs 59.9%; *P* < 0.001) (Hatoum *et al.*, 2018). Similarly to our study, there was no difference in terms of clinical pregnancies. Overall, the higher rates of early pregnancy loss reported with AC might be explained by defective placentation. In addition to early pregnancy loss, AC has also been associated to adverse obstetrical and perinatal outcomes. A retrospective study of 9726 singletons born after FET, of which 6297 NC, 1983 SC and 1446 AC showed that AC was associated with a higher risk of hypertensive disorders during pregnancy, postpartum hemorrhage, post-term birth and macrosomia compared to SC and NC (Ginström Ernstad *et al.*, 2019). Similarly, a retrospective cohort study of FET, of which 29 760 performed with NC and 75 474 performed with AC found increased risks of hypertensive disorders of pregnancy and placenta accreta compared to NC (Saito *et al.*, 2019). Moreover, risk factors associated to early pregnancy loss after FET reported in previous literature were confirmed in our study: maternal age, history of early pregnancy loss and ovulation disorder. The rate of blastocyst transfers was significantly higher in the AC group (40.9% for AC vs 26.3% for NC vs 38.1% for SC, respectively, *P* < 0.05), but was not significantly associated to early pregnancy loss in multivariable analysis.

A real or relative deficiency in progesterone during early pregnancy might increase the risk of early pregnancy loss. Indeed, progesterone plays an essential role in the secretory transformation of the endometrium to enable synchronization with embryonic development and maintain pregnancy. An insufficient progesterone concentration at the time of implantation or at the start of pregnancy may result in early pregnancy loss or defective placentation. Progesterone also reduces the contractility of the myometrium (Hill *et al.*, 1990). For FET with AC, sufficient exogenous supplementation with progesterone is mandatory since no functional corpus luteum is present. In our study, luteal phase support by progesterone was homogeneous overall. In our study, the differences between centers concerning the dose of progesterone used did not affect the results. Indeed, no significant difference was observed in early pregnancy loss rates between the nine centers, whatever the dose used, which ranged from 400 to 800 mg per day (micronized progesterone by vaginal route in all centers). The dosage and route of administration of progesterone supplementation is currently discussed. The 2010 Cochrane meta-analysis (Glujovsky *et al.*, 2010) and the 2017 meta-analysis of Mackens *et al.* (2017) agree that there is no superior route of administration. A more recent American prospective study (Devine *et al.*, 2018) suggests that the intramuscular route results in fewer early pregnancy loss and hence more live births. Pharmacokinetics is known to differ between the different routes (Miles *et al.*, 1994). Although several studies (Miles *et al.*, 1994; Kofinas *et al.*, 2015; Yovich *et al.*, 2015) have investigated the association between progesterone levels on the day of embryo transfer and the outcome of FET in AC, no clear guideline concerning a progesterone level cut-off exists so far. Serum progesterone levels <10 ng/ml on transfer day were reported to be significantly associated to lower pregnancy rates (*P* = 0.04) and live birth rates (*P* = 0.01) (Cédrin-Durnerin *et al.*, 2019), which is consistent with findings of Gaggiotti-Marre *et al.* (2019). Furthermore, increasing the dose of progesterone administered in case of levels <10 ng/ml did not improve pregnancy rates. Recently, serum progesterone levels <8.8 ng/ml on the day of embryo transfer was associated to lower ongoing pregnancy rate in both autologous and donated oocyte cycles (Labarta *et al.*, 2021).

The association between serum progesterone levels and outcomes in autologous FET using AC have also been described whether progesterone was administered using the vaginal route (Yovich *et al.*, 2015; Alsbjerg *et al.*, 2018; Basnayake *et al.*, 2018) or the intramuscular route (Kofinas *et al.*, 2015; Liu *et al.*, 2018; Boynukalin *et al.*, 2019). Moreover, a prospective study combining vaginal and rectal progesterone in AC cycles reported a non-linear relationship between serum progesterone levels and ongoing pregnancy rates, and that there might be an upper threshold above which pregnancy rates are decreased (Alsbjerg *et al.*, 2020). The same relationship has been observed in the specific context of oocyte donation (Brady *et al.*, 2014; Labarta *et al.*, 2017; Labrosse *et al.*, 2021). Nonetheless, important interindividual variabilities of serum progesterone levels have been described after either route of progesterone administration and various parameters might affect progesterone levels, thus making it particularly difficult to predict serum concentrations after a given dosage (Nahoul *et al.*, 1993; Brady *et al.*, 2014; Kofinas *et al.*, 2015; Merriam *et al.*, 2015). Notably, large variations of serum progesterone levels were reported despite all women receiving the same AC protocol (Yovich *et al.*, 2015). Ultimately, no consensus exists on the optimal threshold for progesterone levels. Systematic assessment of serum progesterone levels during AC, so as to adapt supplementation by vaginal progesterone or to use other routes of administration (subcutaneous, oral) in order to avoid low progesterone levels in AC appears essential (Labarta *et al.*, 2017; Cédrin-Durnerin *et al.*, 2019; Alsbjerg *et al.*, 2020). Furthermore, the use of dydrogesterone might also be an option. A prospective cohort study reported that the addition of dydrogesterone to vaginal progesterone compared to vaginal progesterone only was significantly associated to a lower risk of early miscarriage and to a significantly higher live birth rate in multivariate analysis (Vuong *et al.*, 2021). A retrospective study of 9726 singletons born after FET, of which 6297 NC, 1983 SC and 1446 AC showed that AC was associated with a higher risk of hypertensive disorders during pregnancy, postpartum hemorrhage, post-term birth and macrosomia compared to SC and NC (Ginström Ernstad *et al.*, 2019). Similarly, a retrospective cohort study of FET, of which 29 760 performed with NC and 75 474 performed with AC found increased risks of hypertensive disorders of pregnancy and placenta accreta compared to NC (Saito *et al.*, 2019).

In all, AC was the most used protocol in the nine centers, in line with current practice worldwide. This might be explained by the fact that AC has the advantage of enabling flexibility and simple regulation of the number and timing of transfers for IVF centers. As our study reports that AC is associated to higher rates of early pregnancy loss, therapeutic adaptation and the combination of routes of administration and/or monitoring of serum progesterone in patients undergoing FET with AC might be options to avoid the excess of early pregnancy loss. Routine measurements of serum progesterone levels in AC should be considered. Future studies exploring the efficiency of different routes of progesterone administration and prospective randomized studies analyzing strategies to obtain optimal serum progesterone levels in AC are warranted.

## Data availability

The data underlying this article will be shared on reasonable request to the corresponding author.

## Authors’ roles

L.V. was responsible for data collection, data analysis and writing of the first draft of the manuscript. F.P. was responsible for study design and data collection. G.P-B., N.C., J.G., N.A., J.P.A., C.R., P.E.B., E.M.d’A. and I.C.-D. contributed to the execution of the study and critical discussion. J.L. was responsible for the execution and manuscript drafting. N.M. was responsible for study design, execution, data analysis and manuscript drafting.

## Funding

None.

## Conflict of interest

The authors declare no conflicts of interest in relation to this work. G.P.-B. declares consulting fees from Ferring, Gedeon-Richter, Merck KGaA, Theramex, Teva; Speaker’s fees or equivalent from Merck KGaA, Ferring, Gedeon-Richter, Theramex, Teva. N.C. declares consulting fees from Ferring, Merck KGaA, Theramex, Teva; Speaker’s fees or equivalent from Merck KGaA, Ferring. C.R. declares a research grant from Ferring, Gedeon-Richter; consulting fees from Gedeon-Richter, Merck KGaA; Speaker’s fees or equivalent from Merck KGaA, Ferring, Gedeon-Richter. E.M.d’A. declares Speaker’s fees or equivalent from Merck KGaA, MSD, Ferring, Gedeon-Richter, Theramex, Teva. I.C-D. declares Speaker’s fees or equivalent from Merck KGaA, MSD, Ferring, Gedeon-Richter, IBSA. N.M. declares a research grant from Merck KGaA, MSD, IBSA; consulting fees from MSD, Ferring, Gedeon-Richter, Merck KGaA; Speaker’s fees or equivalent from Merck KGaA, MSD, Ferring, Gedeon-Richter, Teva, Goodlife, General Electrics.

## Supplementary Material

hoac007_Supplementary_Table_S1

hoac007_Supplementary_Table_S2
