# Supplementary material for: Impact of endometrial preparation on early pregnancy loss and live birth rate after frozen embryo transfer: a large multicenter cohort study (14 421 frozen cycles)
Source: Hum Reprod Open. 2022 Feb 15;2022(2):hoac007. doi: 10.1093/hropen/hoac007 (PMC8902977; doi:10.1093/hropen/hoac007)
Supplement: hoac007_Supplementary_Table_S1 [file hoac007_supplementary_table_s1.docx]

|  | **Total cycles** | **Artificial cycle (AC)** | **Natural cycle (NC)** | **Stimulated cycle (SC)** | *P* |
| --- | --- | --- | --- | --- | --- |
| **Tubal infertility (%)** | 1710 | 940 (11.5) | 279 (8.9) | 491 (15.6) | *<0.005* |
| **Idiopathic infertility (%)** | 983 | 505 (6.2) | 295 (9.4) | 183 (5.8) | *<0.005* |
| **Mixed infertility (%)** | 2152 | 1276 (15.7) | 400 (12.8) | 476 (15) | *<0.005* |
| **Masculine infertility (%)** | 1841 | 911 (11.2) | 535 (17.1) | 395 (12.5) | *<0.005* |
| **Single embryo transfer (%)** | 2126 (55.3) | 1233 (55.7) | 521 (64.2) | 372 (45.5) | *< 0.05* |
| **Blastocyst (%)** | 1723 (38.0) | 1053 (40.9) | 292 (26.3) | 378 (38.1) | *< 0.05* |
| **Cleavage stage embryo (%)** | 1642 (62.0) | 866 (59.1) | 452 (63.7) | 324 (61.9) | *< 0.05* |

**Supplementary Table SI**

Patient characteristics and number of frozen-thawed embryo transfers according to endometrial preparation protocol
