# Supplementary material for: Impact of endometrial preparation on early pregnancy loss and live birth rate after frozen embryo transfer: a large multicenter cohort study (14 421 frozen cycles)
Source: Hum Reprod Open. 2022 Feb 15;2022(2):hoac007. doi: 10.1093/hropen/hoac007 (PMC8902977; doi:10.1093/hropen/hoac007)
Supplement: hoac007_Supplementary_Table_S2 [file hoac007_supplementary_table_s2.docx]

|  | **Early pregnancy loss** | | **Univariable analysis** |  |
| --- | --- | --- | --- | --- |
|  | **N** | **%** | **Odds Ratio (CI 95%)** | p |
| **Centre** |  |  |  |  |
| A | 253 | 31.0 |  | - |
| B | 109 | 27.3 | 0.83 (0.64–1.09) | 0.18 |
| C | 155 | 35.1 | 1.21 (0.94–1.54) | 0.13 |
| D | 115 | 32.9 | 1.09 (0.83–1.42) | 0.53 |
| E | 137 | 28.8 | 0.90 (0.70–1.52) | 0.40 |
| F | 136 | 34.3 | 1.16 (0.90–1.50) | 0.24 |
| G | 108 | 32.9 | 1.09 (0.83–1.43) | 0.53 |
| H | 94 | 34.9 | 1.20 (0.89–1.60) | 0.23 |
| I | 103 | 28.0 | 0.87 (0.66–1.14) | 0.30 |
|  |  |  |  |  |

**Supplementary Table SII**: Early pregnancy loss per pregnancy with HCG > 100 IU/mL according to centers
